# Investigation on the Microstructure and Mechanical Properties of 304 Stainless Steel Joints by Underwater Local Dry Laser Welding

**DOI:** 10.3390/ma19091723

**Published:** 2026-04-23

**Authors:** Xiaodong Zhang, Fangjie Cheng, Yingchao Feng, Jinping Liu, Zhuyuan Li, Yehua Wu, Ke Han, Qianxing Yin

**Affiliations:** 1 School of Materials Science and Engineering, Tianjin University, Tianjin 300072, China; zhangxiaodong@cni23.com (X.Z.); jinpingliu1990@163.com (J.L.); 2 Key Laboratory for Highly Efficient and Intelligent Welding of China National Nuclear Corporation, China Nuclear Industry 23 Construction Co., Ltd., Beijing 101300, China; fengcni23@163.com (Y.F.); lizhuyuan87@126.com (Z.L.); wuyehua@cni23.com (Y.W.); 3 Nuclear Industry Research and Engineering Co., Ltd., Beijing 101300, China; 4 School of Materials Science and Engineering, Jiangsu University, Zhenjiang 212013, China; hanke@ujs.edu.cn; 5 School of Power and Mechanical Engineering, Wuhan University, Wuhan 430072, China; yinqianxing@whu.edu.cn

**Keywords:** underwater local dry laser welding, 304L stainless steel, microstructure, mechanical properties

## Abstract

In order to verify the feasibility of in situ repair of underwater local dry laser welding (ULDLW) on nuclear power reactor components, this work investigates the microstructure and mechanical properties of 304L austenitic stainless steel repaired by ULDLW using ER308L filler metal. Comprehensive comparison would be made between the ULDLW and conventional in-air laser welding to evaluate their applicability. The results demonstrate that the rapid cooling rate inherent to the underwater environment significantly influences solidification behavior and microstructural evolution. The weld metal (WM) solidifies in the ferritic–austenitic (FA) mode, with an increased proportion of lathy δ-ferrite at the expense of skeletal morphology compared to the in-air welds. Electron backscatter diffraction (EBSD) analysis reveals the substantial grain refinement in underwater welds, with average grain sizes of 39.4 μm versus 47.3 μm for in-air weld bead, accompanied by a higher fraction of low-angle grain boundaries (LAGBs). These microstructural modifications yield superior mechanical properties: underwater weld bead exhibits ultimate tensile strength (UTS) of 685.6 MPa, elongation of 57.5%, and impact toughness of 22.6 J, significantly exceeding the corresponding values for in-air welds (663.9 MPa, 51.8%, and 18.6 J, respectively). Fractographic analysis confirms ductile fracture mechanisms in both conditions. The enhanced performance is attributed to grain refinement strengthening via the Hall–Petch relationship and the increased LAGBs fraction, which impedes dislocation motion and crack propagation.

## 1. Introduction

Ultra-low carbon 304L stainless steel (C ≤ 0.01%) has gained significant attention in the nuclear power field due to its outstanding corrosion resistance and weldability. The diminished C content serves to minimize both the precipitation of Cr-carbides due to welding as well as its susceptibility to intergranular-type corrosion. Consequently, it has become a predominant material for pressure boundary as well as many core internal components in light water reactors [1,2,3]. Nevertheless, during long-term service, aging-related degradation of nuclear facilities, particularly underwater reactor components exposed to high-temperature and high-radiation environments, can lead to various forms of damage [4,5].

Ensuring operational safety requires effective in situ repair techniques for submerged reactor components, an urgent engineering challenge [6]. Underwater arc welding is widely used for on-site repairs [7,8], but it suffers from susceptibility to water depth, turbidity, and flow; produces broad heat-affected zones and debris; and often involves instability and safety hazards [8]. In contrast, laser beam welding offers a narrow heat-affected zone, non-contact processing, and automation compatibility, minimizing thermal distortion, reducing contamination, and improving efficiency [9,10,11,12]. These advantages make underwater laser beam welding particularly attractive for remote, precise repair of critical reactor internals under harsh conditions.

Research on underwater laser beam welding focuses on wet and local dry approaches. Guo et al. [12] studied laser-induced plasma evolution and weld quality at varying depths, finding that water depths above 7 mm impede the process. Wen et al. [13] employed a pre-placed protective material on the base metal during wet underwater laser beam welding, thereby extending the achievable water depth to 20 mm.

To ensure high-quality welds at conventional depths, water must be fully excluded from the laser-irradiated region, prompting development of local dry techniques. Fu et al. [14] successfully generated a stable local dry cavity around the weld zone using a gas flow rate of 50 L/min, producing joints with tensile strength and impact toughness comparable to those obtained from in-air laser welding. Wang et al. [15] reported that the use of pure nitrogen as the shielding gas, in contrast to other gases, led to enhanced corrosion resistance in the weld region. Guo et al. [16] systematically investigated the influence of various process parameters on weld morphology and mechanical properties in underwater laser wet welding using a custom-designed double-layer gas curtain nozzle. Their microstructural analysis revealed the presence of lathy ferrite and the replacement of some equiaxed crystals by columnar dendrites. Zhang et al. [17] examined the correlation between shielding conditions within the local dry cavity (shielding gas: Ar) and weld quality, demonstrating that the stability of the cavity serves as a critical indicator for process monitoring during underwater laser beam welding. In a subsequent study, Guo et al. [18] further characterized the surface morphology, microstructure, and mechanical properties of underwater laser beam welding joints produced under controlled parameter conditions using a double-layer gas protective cover.

In view of the results of the aforementioned studies research, this work employed an optimized double-layer air curtain drainage cover to perform local dry underwater laser beam welding on 304L stainless steel. A comparative analysis is conducted with laser beam welding performed in in-air and underwater environment, aiming to elucidate the microstructural evolution and mechanical properties of 304L weld beads produced by underwater laser beam welding.

Following the Introduction, this work is organized as follows: Section 2 describes the materials, groove design, ULDLW equipment, and experimental procedures for microstructural and mechanical characterization. Section 3 presents the results and discussion, covering the macromorphology of weld beads, microstructural evolution, and the influence of the underwater environment on grain refinement. It also evaluates the mechanical properties—tensile strength, elongation, and impact toughness—and correlates them with the microstructural features. Finally, Section 4 summarizes the main conclusions of this work.

## 2. Materials and Methods

**Figure 1:**
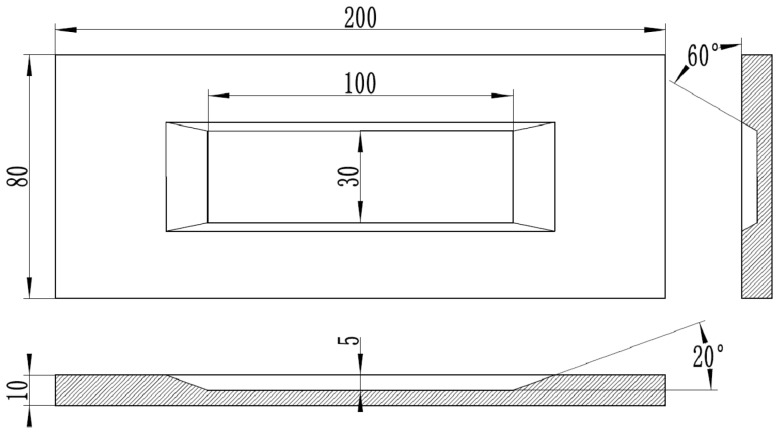
The size of BM and filler groove.

The base material (BM) utilized in this work is 304L austenitic stainless steel, which is widely employed in industrial applications such as reactor and storage tank manufacturing, attributed to its favorable combination of mechanical properties and corrosion resistance. The BMs are provided in a solution-treated condition and machined into plates with a size of 200 mm × 80 mm × 10 mm. Prior to welding, an isosceles trapezoidal groove (100 mm × 30 mm × 5 mm), with a bevel angle of 20° and 60° between the bevel face and the groove bottom, was fabricated at the center of each plate, as illustrated in Figure 1.

**Figure 2:**
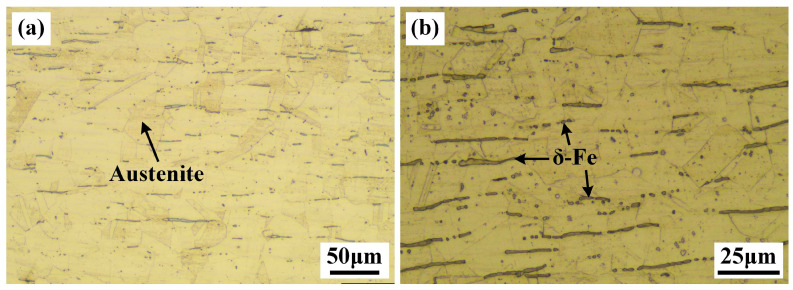
The microstructure of 304L stainless steel: (**a**) low-power microstructure; (**b**) high-power microstructure.

The groove was polished to smooth finish. The microstructure of BM is shown in Figure 2, consisting of austensite and δ ferrite. δ ferrite exhibits an elongated stripe morphology aligned parallel to the rolling direction and distributed in a multilayered manner, a characteristic attributable to the hot rolling treatment experienced by the BM. During hot rolling, the BM undergoes substantial plastic deformation, resulting in the elongation of austenite grains and the formation of a fibrous structure along the rolling direction. Subsequently, ferrite formed via the transformation of austenite preferentially nucleates at austenite grain boundaries, thereby inheriting the elongated shape and maintaining its alignment parallel to the rolling direction. The chemical composition of BM is presented in Table 1. In order to guarantee the mechanical properties and corrosion resistance of the weld bead, the grade ER308L wire is selected and its chemical composition is shown in Table 1.

**Figure 3:**
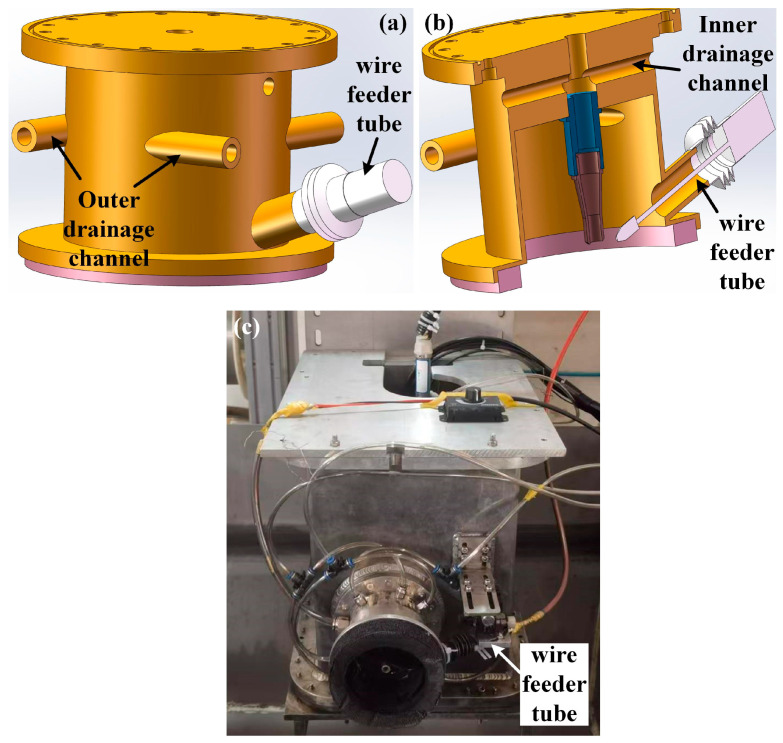
Schematic diagram (**a**,**b**) and physical image (**c**) of the drainage cover.

The ULDLW experiments were conducted using a dual-layer gas curtain drainage cover, as schematically shown in Figure 3. The RFL-C3000S fiber laser (Wuhan Raycus Fiber Laser Technologies Co., Ltd., Wuhan, China) with a maximum output power of 3 kW and an operating wavelength of 1080 nm was employed in this study. Before welding, the plate surfaces were mechanically ground using abrasive paper, subsequently polished, and degreased with alcohol to remove surface contaminants and oxides. The welding parameters applied in both in-air and underwater environments are summarized in Table 2. The water depth was 200 mm.

**Figure 4:**
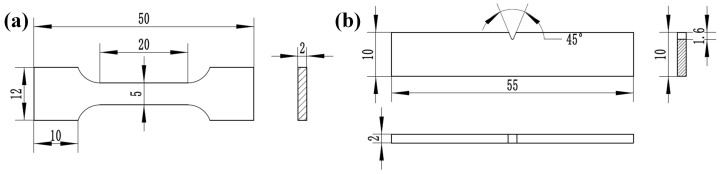
The dimension of tensile and impact specimens: (**a**) tensile specimens; (**b**) impact specimens.

Metallographic etching was conducted using aqua regia prepared with a volumetric HNO_3_:HCl ratio of 1:3. The macrostructure of the weld bead was examined using a stereomicroscope (Nikon SMZ25, Nikon Corporation, Tokyo, Japan), while microstructural characterization was performed via optical microscopy (LEICA DM2500M, Leica Microsystems GmbH, Wetzlar, Germany). The chemical composition of the WM was detected by the Electron Probe Microanalyzer (EPMA, JXA-iHP200F, JEOL Ltd., Tokyo, Japan). EBSD analysis was carried out using a TSL Hikari Super detector to assess the GB characteristics. Tensile tests were conducted at room temperature on a universal testing machine (CIMACH DDL100, Changchun Research Institute for Mechanical Science Co., Ltd., Changchun, China) at a crosshead speed of 2 mm/min. The tensile specimens (three specimens for each weld bead) were dog-bone shaped, with a length of 50 mm and a cross-sectional width of 5 mm, as illustrated in Figure 4a. Fracture surfaces of the tensile specimens were subsequently examined using scanning electron microscopy (FEI Nova NanoSEM 450, SEM, FEI Company, Hillsboro, OR, USA). Impact testing (three specimens for each weld bead) was performed on a metal pendulum impact tester (NI300, NCS Testing Technology Co., Ltd., Beijing, China), and the dimensions of the impact specimens are presented in Figure 4b.

## 3. Results and Discussion

### 3.1. Macromorphology of the Weld Beads

**Figure 5:**
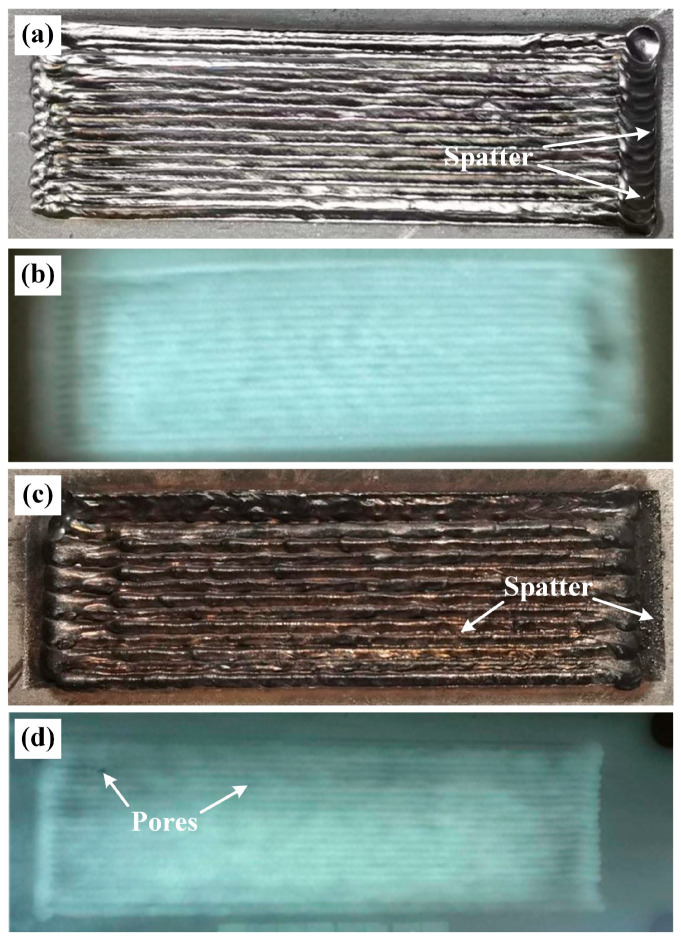
Macromorphology and X-ray detection images of the weld beads under different welding conditions: (**a**,**b**) underwater; (**c**,**d**) in-air.

Figure 5 shows the surface morphology of weld beads in the underwater and in-air environments. As can be seen from Figure 5a, the surface of the weld bead in underwater environment is well-formed, appearing bright silver in color. The weld is uniform in width, without defects such as lack of fusion, porosity, or cracks. No welding defects are found in Figure 5b, confirming the aforementioned results. However, the weld beads (Figure 5c) in in-air environment appear dark gray, with severe oxidation in the surface. In addition, a large amount of spatters are formed in the appearance of the weld bead. Furthermore, the pores are found within the weld bead, which indicates that the weld beads obtained in the in-air environment presents poor formation quality. The well-formed weld beads in the underwater environment are decided by the excellent protective effect and rapid cooling rate by the water [12]. On the one hand, the ULDLW creates a completely dry space inside the drainage cover by the protective gas (e.g., Ar) and isolates the direct action of water and air on the molten pool, further optimizing the metallurgical effect. On the other hand, the molten pool solidifies rapidly by the extremely high thermal conductivity of water, preserving the regular ripples. Moreover, the rapid cooling effect of water significantly increases the surface tension of the molten metal, promoting the molten pool to spread into full and uniform arc shape, effectively avoiding excessive flow or collapse that is prone to occur in air environment due to the slow cooling. As a result, these factors work to obtain the sound weld beads in the underwater environment.

**Figure 6:**
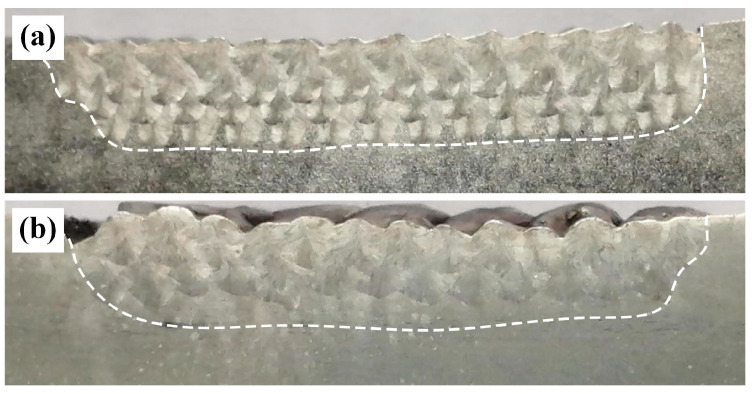
Cross-section macrostructure of the weld beads under different welding conditions: (**a**) underwater; (**b**) in-air.

Figure 6 shows the macroscopic morphologies of the weld beads in the underwater and in-air environments. As is known from Figure 6, the number of weld layers is three, with clear boundaries between the different layers. The fusion interface between the weld layer and BM exhibits a distinct wavy pattern, and no defects such as porosity or cracks are present, indicating the formation of good metallurgical bond.

### 3.2. Microstructure of the Weld Beads

**Figure 7:**
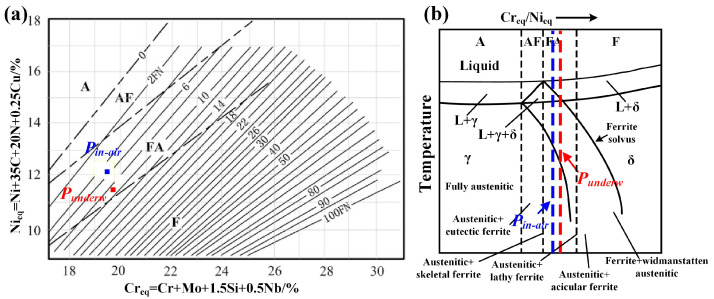
The microstructure transformation of WM in the underwater condition: (**a**) Schaeffler diagram; (**b**) pseudo-binary sections of the Fe–Cr–Ni ternary system [19].

The solidification behavior of the welded joint was initially examined. In austenitic stainless steel welds, the solidification mode is primarily governed by the ratio of the chromium equivalent [Cr]_eq_ to the nickel equivalent [Ni]_eq_, both of which are derived from the chemical compositions of the BM and filler metal. Consequently, the solidification mode and sequence can be predicted using the Schaeffler diagram in conjunction with pseudo-binary sections of the Fe–Cr–Ni ternary system [19]. In order to ensure the [Cr]_eq_ and [Ni]_eq_ of the WM, the chemical composition of the WM in the underwater and in-air environment is shown in Table 3. Based on the calculations of the [Cr]_eq_ and [Ni]_eq_, the solidification mode of the WMs is determined to be FA mode, corresponding to point P_underw_ and P_in-air_ in Figure 7a. Based on these findings, the solidification sequence of the WM can be described as L → L + δ → L + δ + γ → δ + γ → γ, indicating that solidification initiates with the formation of primary δ-ferrite, as illustrated in Figure 7b. The FA solidification mode is known to reduce susceptibility to solidification cracking in the weld metal. Consistently, radiographic inspection revealed no cracks in the weld bead.

**Figure 8:**
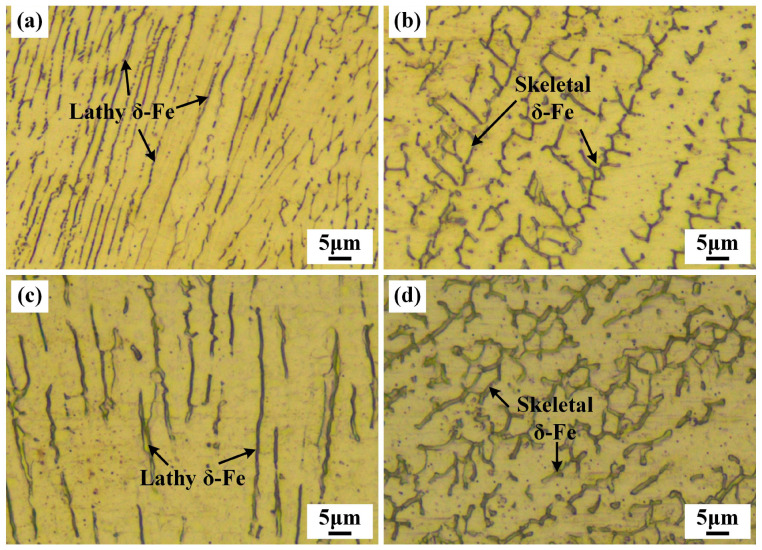
The WM microstructure in the weld beads with different environments: (**a**,**b**) underwater; (**c**,**d**) in-air.

Figure 8 illustrates the morphology and distribution of the WM microstructure with different welding environments. The microstructures of all the WM mainly comprise austenite (light color) and δ ferrite (deep color), which correspond to the obtained results in Figure 7. For the weld beads in the in-air environment, the δ-ferrite primarily exhibited a skeletal morphology, with a minor presence of lathy δ phase. The microstructure of the WM in the underwater weld bead illustrates that both skeletal and lathy δ-ferrite morphologies are observed. However, the proportion of lathy δ-ferrite increased noticeably compared to that in the in-air weld bead. In laser welding processes, the microstructural evolution of the weld bead is governed by the thermal cycle experienced during solidification. Jia et al. found that the cooling rate of the weld in the underwater and in-air environments is 3657 °C/s and 2281°C/s in the high temperature range (1060 °C→800 °C), respectively [20]. The higher cooling rate results in a steeper thermal gradient. According to Kristensen [21], under moderate cooling rates, the δ → γ transformation proceeds with diffusion control, favoring the formation of skeletal δ-ferrite. In contrast, under rapid cooling conditions, atomic diffusion is inhibited, and the reduced diffusion distance promotes the development of the refined, closely spaced lathy δ-ferrite microstructure. The accelerated cooling rate induced by the underwater environment thus accounts for the increased content of lathy δ-ferrite in the FZ of underwater laser welded joints relative to their in-air counterparts.

**Figure 9:**
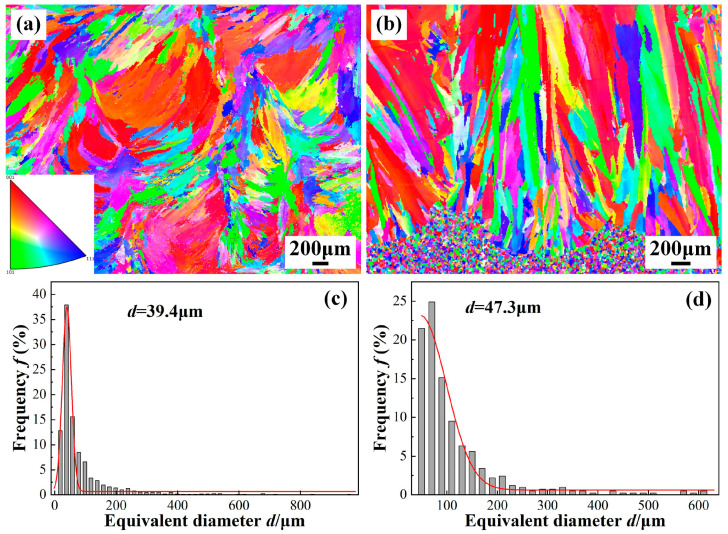
IPFs and grain size of the WM under different welding conditions: (**a**,**c**) underwater; (**b**,**d**) in-air.

The EBSD was performed to analyze the grain morphology and the grain size. Figure 9 displays grain orientation maps and grain size of the underwater and in-air WM. Both of WMs in the underwater and in-air weld beads exhibit the typical columnar crystal morphology. Compared with the underwater WM, the columnar crystal of the in-air WM is more pronounced, and the orientation of grain growth is stronger. The grain sizes of the WMs are measured and the result is exhibited in Figure 9b. The average grain size of the WMs is 39.4 μm and 47.3 μm, respectively. Due to the extremely rapid cooling rate of underwater environment, grain growth in the WM is suppressed, resulting in a finer grain size in the weld bead in underwater environment. Similar conclusions were drawn by Fu et al. [14,19].

**Figure 10:**
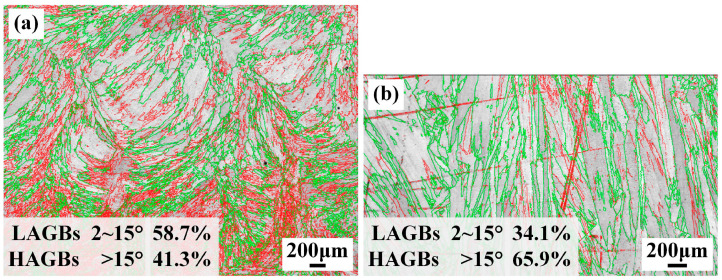
GBs distribution of the WZ under different welding conditions: (**a**) underwater; (**b**) in-air.

**Figure 11:**
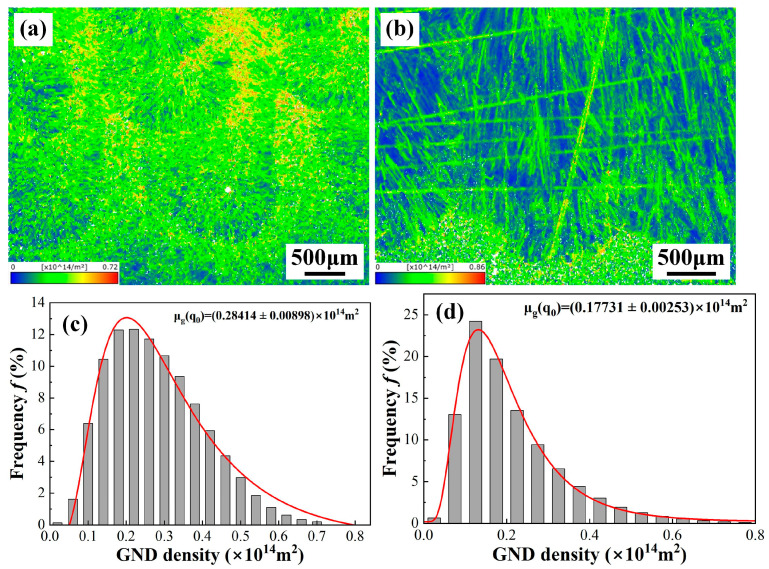
The calculated mean GND density distributions of the WZs in the different environments: (**a**,**c**) underwater; (**b**,**d**) in-air.

GBs are generally categorized into two types based on misorientation angles: LAGBs (2~15°) and high-angle grain boundaries (HAGBs, >15°) [22]. Figure 10 presents the frequency distribution of LAGBs and HAGBs in the welded joints. The WZ of the underwater weld bead exhibits a markedly higher proportion of LAGBs, whereas the in-air WM predominantly consisted of HAGBs. It indicates that the cooling rate may have an impact on the GB character of the WM. To quantitatively investigate the dislocation density of the WMs, Yan et al. pointed out that the kernel average misorientation (KAM) method was adopted to determine the local misorientation by the EBSD orientation data [23]. Figure 11 shows the calculated mean geometrically necessary dislocation (GND) density distributions of the WZs. The mean GND density of the WM in the underwater environment is 2.84 × 10^13^ m^2^, higher than that of the in-air WM. Das et al. [24] reported that an increase in cooling rate caused an increase in LAGBs during the welding process. Therefore, high content of LAGBs are formed in the WM of the weld bead in the underwater environment. In addition, the increased fraction of LAGBs within the WZ indicates an elevated dislocation density in the corresponding grains, which contributes to enhanced mechanical strength by inhibiting crack initiation and propagation [25]. The statistical results of GND density in the WMs also prove the above analysis. Moreover, LAGBs are composed of dislocation arrays with lower interfacial energy, typically resulting in reduced atomic diffusion rates compared to HAGBs. As reported by Laleh et al. [26], the introduction of LAGBs into metallic microstructures can effectively suppress the undesirable intergranular degradation mechanisms, including corrosion, embrittlement, and fracture. Zhang et al. [27] also demonstrated that LAGBs possess superior resistance to intergranular cracking compared to HAGBs. Moreover, Fan et al. confirmed that the introduction of high-density LAGBs into 304 stainless steel via warm-rolling resulted in an exceptional combination of strength, ductility, and toughness [28]. Therefore, the formation of LAGBs is conducive to improving the mechanical properties in the underwater weld bead.

### 3.3. Mechanical Properties of the Weld Beads

**Figure 12:**
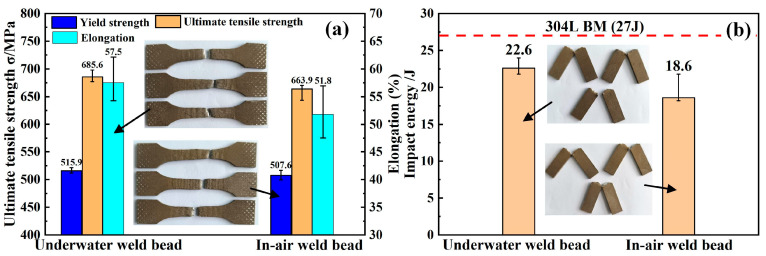
Mechanical properties of underwater and in-air weld beads: (**a**) tensile properties; (**b**) impact energy.

The tensile strength and impact toughness of in-air and underwater weld beads were tested and the obtained results are shown in Figure 12. The laser welding process released the compressive stress of the cold-rolled 304L stainless steel in the WM, causing the decrease in strength. In addition, the formation of the coarse columnar grains also weakens the tensile strength further. Consequently, all tensile samples fracture in the WM. The yield strength, UTS and elongation of the underwater weld bead reach 515.9 MPa, 685.6 MPa and 57.5%, significantly higher than that of the in-air weld bead (507.6 MPa, 663.9 MPa and 51.8%). The advantage in mechanical properties primarily depends on the grain size of the WM in the underwater weld bead. As known from Figure 9, the equivalent diameter of the underwater WM is lower than that of the in-air WM. According to the Hall–Petch relationship [29], the yield strength of metallic materials increases with decreasing grain size. The phenomenon is attributed to the enhanced impediment of GBs to dislocation motion, wherein dislocations encounter greater resistance within finer grains, thereby elevating the material’s strength. Furthermore, under the external deformation, grain refinement promotes more uniform plastic deformation by mitigating localized stress concentrations, which consequently improves the overall ductility and plastic deformation capacity of the material [30]. The synergistic enhancement accounts for the superior combination of strength and ductility observed in underwater weld beads compared to those fabricated in an air environment.

Furthermore, the impact energy of the underwater weld bead is about 22.6 J, 83.7% of the BM (27 J). However, the impact energy of the in-air weld bead (18.6 J) is lower than that of the underwater weld bead. Sun et al. [31] reported that the existence of the δ-ferrite in 304 NG stainless steel joint deteriorated the toughness of WM. Compared with the BM, the fast cooling rate contributes to forming more δ-ferrite in the WM. Therefore, the WM exhibits poor toughness. Mamedipaka et al. reported that the lower cooling rate from the high heat input facilitated the formation of δ-ferrite, which led to a higher fraction of δ-ferrite [32]. In this work, the cooling rate in the underwater environment is far higher than that of the in-air environment. Thus, compared with the in-air WM, the content of δ-ferrite in the underwater WM is relatively lower. In addition, Lezaack et al. [33] pointed out that fine-grain microstructure could significantly enhance the impact toughness of aluminum alloy. Based on the results in Figure 9, the weld bead in the underwater environment shows finer grains. As a result, the toughness of the underwater weld bead is superior to that of the in-air weld bead.

**Figure 13:**
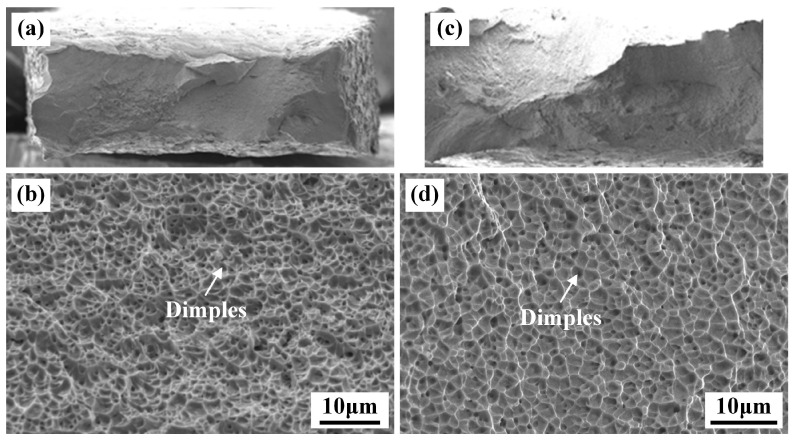
Fractography microstructure of the tensile specimens under different welding conditions: (**a**,**b**) underwater; (**c**,**d**) in-air.

SEM was employed to examine the fracture surfaces of the tensile specimens, with the resulting fractographs presented in Figure 13. The fracture surface of the underwater tensile specimen is characterized by a distribution of dimples and microvoids, indicative of the typical ductile fracture mechanism. As shown in Figure 13c,d, the fracture surface morphology of the in-air specimen closely resembled that of the underwater specimen, suggesting a similar ductile fracture mode.

## 4. Conclusions

In this work, 304L stainless steels are repaired with ER308L filler by in-air laser welding and ULDLW. The microstructure and mechanical properties of the weld beads obtained by the in-air and underwater laser welding are investigated. The following conclusions are drawn as follows:

(1) The underwater environment significantly influences the solidification behavior and microstructural evolution of the WM. The solidification mode of the underwater weld bead is determined to be FA mode, with a solidification sequence of L → L + δ → L + δ + γ → δ + γ → γ, initiating with primary δ-ferrite formation. The accelerated cooling rate induced by the underwater environment promotes the transformation from skeletal to lathy δ-ferrite morphology. Compared with the in-air WM, the extremely high cooling rate resulting from the underwater environment inhibits the formation of δ-ferrite, which led to a lower fraction of δ-ferrite in the underwater WM.

(2) Grain refinement is achieved in underwater laser welds due to the rapid cooling rate, with an average grain size of 39.4 μm compared to 47.3 μm in in-air welds. The refinement is accompanied by a higher fraction of LAGBs in the underwater WM, which is attributed to the increased cooling rate. The enhanced LAGBs fraction indicates elevated dislocation density and contributes to improved mechanical properties by inhibiting crack initiation and propagation.

(3) The weld beads prepared by ULDLW exhibit superior mechanical properties compared to their in-air counterparts, with ultimate tensile strength of 685.6 MPa and elongation of 57.5%, versus 663.9 MPa and 51.8% for in-air welds. The impact toughness of underwater welds (22.6 J) is also higher than that of in-air welds (18.6 J). These enhancements are primarily attributed to grain refinement and the increased proportion of LAGBs, which collectively improve strength and ductility through the Hall–Petch relationship. The excellent mechanical properties of the weld bead by the ULDLW confirm the feasibility and application of in situ repairing 304L stainless steel.

## Figures and Tables

**Table 1:** Chemical compositions of BM and filler wire (wt.%).

Material	C	Mn	Si	S	P	N	Ni	Cr
BM	0.019	1.72	0.42	0.001	0.039	0.05	8.0	18.0
ER308L	0.06~0.15	1.40~1.85	0.80~1.15	0.025	0.025	0.50	0.15	0.15

**Table 2:** The ULDLW process parameters.

Laser Power (W)	Defocus d_1_ (mm)	WELDING Speed v_1_ (mm/min)	Wire Feed Speed v_2_ (mm/min)	Overlap Value d_2_ (mm)	Gas Flow Rate Q (L/min)	
					Inner Air Vent (N_2_)	Outer Air Vent (Ar)
2000	90	19.7	1500	50%	40	40

**Table 3:** Chemical composition of the WMs in the underwater and in-air environment (wt.%).

	Alloy Elements Content (%)	Point 1	Point 2	Point 3	Average Content (%)	[Cr]_eq_	[Ni]_eq_	[Cr]_eq_/[Ni]_eq_
WM in underwater environment (P_underw_)	C	0.023	0.019	0.032	0.025	19.711	11.455	1.721
	Si	0.409	0.461	0.403	0.424			
	Mn	1.504	1.445	1.337	1.429			
	P	0	0.015	0.017	0.011			
	S	0.008	0	0	0.003			
	Ni	8.953	10.035	8.656	9.215			
	Cr	19.053	19.048	18.842	18.981			
	Co	0.068	0.131	0.109	0.103			
	Mo	0.068	0.115	0.075	0.086			
	Nb	0.044	0	0	0.015			
	Cu	0.041	0.013	0	0.018			
	Ta	0	0	0.083	0.028			
	V	0.03	0.056	0.051	0.046			
	N	0.188	0.015	0	0.068			
WM in in-air environment (P_in-air_)	C	0.024	0.047	0.068	0.046	19.47	12.128	1.605
	Si	0.463	0.121	0.125	0.236			
	Mn	1.673	0.712	0.787	1.057			
	P	0.032	0.029	0.023	0.028			
	S	0	0.001	0	0.000			
	Ni	9.792	7.963	7.411	8.389			
	Cr	18.439	19.147	19.446	19.011			
	Co	0.129	0.261	0.193	0.194			
	Mo	0.067	0.087	0.098	0.084			
	Nb	0.019	0.006	0.101	0.042			
	Cu	0	0.078	0.03	0.036			
	Ta	0.031	0.076	0.114	0.074			
	V	0.047	0.065	0.092	0.068			
	N	0.109	0.132	0.078	0.106			

## Data Availability

The original contributions presented in this study are included in the article. Further inquiries can be directed to the corresponding author.
